# Impact of Candida Care Bundle Compliance on the Prognosis of Patients With Candidemia: A Multicenter Retrospective Cohort Study With Propensity Score Matching Analysis (2016–2023)

**DOI:** 10.1093/ofid/ofaf790

**Published:** 2025-12-23

**Authors:** Hidemasa Akazawa, Shinnosuke Fukushima, Toshie Higuchi, Tomoko Miyoshi, Yasuhiro Nakano, Koji Iio, Yukinobu Akamatsu, Yuto Haruki, Yoshitaka Iwamoto, Shuichi Tanaka, Shun Fujisato, Soichiro Ako, Hideharu Hagiya

**Affiliations:** Department of Infectious Diseases, Okayama University Hospital, Okayama, Japan; Department of General Medicine, Okayama University Graduate School of Medicine, Dentistry and Pharmaceutical Sciences, Okayama, Japan; Department of Infectious Diseases, Okayama University Hospital, Okayama, Japan; Department of Bacteriology, Okayama University, Graduate School of Medicine, Dentistry and Pharmaceutical Sciences, Okayama, Japan; Department of General Internal Medicine, Okayama Red Cross Hospital, Okayama, Japan; Center for Medical Education and Internationalization, Kyoto University Graduate School of Medicine, Kyoto, Japan; Department of General Medicine, Okayama University Graduate School of Medicine, Dentistry and Pharmaceutical Sciences, Okayama, Japan; Microbiology Division, Clinical Laboratory, Okayama University Hospital, Okayama, Japan; Department of General Medicine, Tottori Municipal Hospital, Tottori, Japan; Department of Pharmacy, Tsuyama Chuo Hospital, Okayama, Japan; Department of General Medicine, NHO Okayama Medical Center, Okayama, Japan; Department of General Medicine, Okayama University Graduate School of Medicine, Dentistry and Pharmaceutical Sciences, Okayama, Japan; Department of Pharmacy, Okayama Rousai Hospital, Okayama, Japan; Department of General Medicine, Okayama University Graduate School of Medicine, Dentistry and Pharmaceutical Sciences, Okayama, Japan; Department of Infectious Diseases, Okayama University Hospital, Okayama, Japan

**Keywords:** candida bundle, candidemia, endophthalmitis, prognosis, propensity score matching

## Abstract

**Background:**

Candidemia is a life-threatening infection with high mortality, and appropriate management is essential to improve patient outcomes. The Candida Care Bundle aims to standardize hospital management for patients with candidemia and reduce mortality.

**Methods:**

This retrospective multicenter cohort study included candidemia cases from 9 hospitals in Japan between 2016 and 2023. Compliance to the Candida Care Bundle was evaluated based on 5 elements: central venous catheter removal within 24 hours, appropriate antifungal therapy, ophthalmologic examination, follow-up blood cultures, and antifungal treatment for ≥2 weeks after clearance. Patients were categorized into high (4–5 items) and low (0–3 items) compliance groups. The primary and secondary outcomes were defined as 30-day survival and the development of endophthalmitis, with propensity score matching used to adjust for potential confounders.

**Results:**

Among 230 patients, 160 (69.5%) were classified into the high compliance group, which exhibited significantly lower 30-day mortality than the low compliance group (8.8% vs 57.1%, *P* < .01). Even after matching, the high compliance group remained independently associated with improved survival (hazard ratio [HR]: 0.15; 95% confidence interval [CI]: .08–.30). *C. albicans* (HR: 1.95; 95% CI: 1.01–3.52) and central line-associated bloodstream infection (HR: 2.63; 95% CI: 1.35–5.12) were associated with the fatal outcome. Endophthalmitis involved 23.6% of the patients, being associated with *C. albicans* (odds ratio [OR]: 8.18; 4.46–19.30) and central line-associated bloodstream infection (OR: 2.69; 1.08–6.70).

**Conclusions:**

Strict compliance to the Candida Care Bundle significantly improves survival, underscoring its importance in candidemia management.

*Candida* species are ubiquitous yeast-like fungi that constitute part of the normal microbiota on human skin, as well as in the gastrointestinal tract, urinary tract, and genital mucosa [1, 2], and are leading fungal pathogens responsible for both superficial and invasive infections [3]. In vulnerable individuals, such as the elderly and immunocompromised patients, *Candida* species can cause serious opportunistic infections [4]. Among progressive Candida infections, candidemia—defined by the presence of *Candida* species in blood—can lead to multi-organ involvement, including endophthalmitis, in approximately 15%–25% of cases [5–8]. Candidemia is associated with a poor prognosis, with reported fatality rates ranging from 24.4% to 62.6% even in developed countries [9–12].

Recent reports have indicated an increased incidence of candidemia during the COVID-19 pandemic [13]. In addition, the emergence of drug-resistant strains such as *Candida auris* and fluconazole-resistant *Candida parapsilosis* has raised global concern over antifungal resistance [14–16]. In response to these developments, the World Health Organization published the Fungal Priority Pathogens List in 2022, categorizing *Candida albicans* and *C. auris* as critical priority pathogens and *C. parapsilosis*, *Candida tropicalis*, and *Candida glabrata* as high-priority pathogens [17, 18].

Sophisticated management of candidemia is essential to improve patient outcomes, which includes the following components: (i) collection of 2 sets of blood cultures; (ii) early and appropriate antifungal therapy; (iii) prompt source control; (iv) removal of central venous catheters (CVCs) within 24 hours of diagnosis; (v) assessment of therapeutic efficacy between days 3 and 5; (vi) ophthalmologic evaluation; (vii) follow-up blood cultures until clearance is confirmed; (viii) optimization of treatment duration; and (ix) oral step-down therapy in patients with favorable clinical response [19–22]. Implementation of these Candida Care Bundle practices has been reported to reduce mortality by ∼10% [19, 20].

Although several multicenter studies have explored patient outcomes in relation to Candida Care Bundle compliance, in-depth analyses stratified by bundle compliance rates remain limited. In this study, we aimed to investigate the impact of bundle compliance on survival among patients with candidemia. To minimize potential confounding factors and improve the validity of intergroup comparisons, propensity score matching was employed.

## METHODS

### Study Design and Settings

This retrospective, multicenter cohort study analyzed data gathered over 8 years (January 2016–December 2023) from 9 hospitals across Okayama, Tottori, and Kagawa Prefectures in Japan. Clinical and microbiological information of patients with candidemia was retrieved from electronic health records following a unified data collection protocol.

### Eligibility Criteria

Patients were eligible for inclusion if they were aged 18 years or older and had at least 1 blood culture positive for *Candida* species. Exclusion criteria included: (1) death prior to antifungal therapy initiation; (2) blood culture results deemed contaminant; (3) death within 3 days of treatment initiation; (4) insufficient clinical or microbiological data; (5) cases in which 2 or more *Candida* species were isolated; and (6) transfer or discharge from the hospital without outcome documentation.

### Definitions

Candidemia was defined by the isolation of *Candida* species from at least 1 blood culture. Because the study was retrospective, contamination was operationally defined as cases in which the treating physician explicitly documented contamination in the medical record or antifungal therapy was not initiated. Otherwise, any isolation of *Candida* species from at least 1 blood culture was considered candidemia. Nosocomial cases were classified as those occurring 48 hours or more after hospital admission [23]. The sources of *Candida* infection in each case were determined from a clinical perspective. CLABSI was assigned when a CVC was in place and no other alternative source was identified. Cases involving recent abdominal surgery, pancreatitis, gastrointestinal perforation or leakage, or an intra-abdominal abscess were classified as having an intra-abdominal source. A urinary tract source, though clinically very rare, was designated only for cases with obstructive uropathy or post-transplant/instrumentation-related infection accompanied by systemic manifestations and corroborated by urine culture results, when no other plausible source was evident. When multiple potential sources coexisted, central line-associated bloodstream infection (CLABSI) was assigned unless compelling evidence indicated another focus. The original Candida Care Bundle comprised the following 9 components: (1) prompt CVC removal within 24 hours; (2) appropriate initial antifungal selection; (3) correct initial dosing; (4) comprehensive ophthalmological evaluations; (5) repeated blood cultures until clearance; (6) clinical review on days 3–5; (7) adjustment to alternative antifungals when necessary; (8) continuation of therapy ≥14 days after clearance; and (9) oral step-down therapy for clinically improving patients [21, 22, 24]. Of these, 5 key interventions were used for the present analysis: (i) early CVC removal; (ii) appropriate initial antifungal selection; (iii) ophthalmological evaluation, (iv) follow-up blood cultures until clearance; and (v) treatment for ≥14 days after clearance. Other components, such as alternative therapy adjustments, precise antifungal dosages, and clinical efficacy assessments, were excluded due to heterogeneity in clinical decision-making. Step-down oral therapy was intentionally excluded given prior reports suggesting its omission may improve outcomes [22]. Accordingly, this component was excluded from the present analysis. Each of the 5 bundle elements contributed 1 point (0–5 total). To facilitate comparison and minimize potential confounding, bundle compliance was dichotomized into high (4–5 items) and low (0–3 items) groups. The threshold of ≥4 completed items was selected a priori to represent adequate implementation of the core bundle, as completion of all 5 elements is sometimes difficult to achieve in real-world clinical practice, whereas adherence to 4 or more components generally reflects sufficient compliance with evidence-based management. In accordance with the 2016 Infectious Diseases Society of America (IDSA) guidelines, appropriate initial antifungal therapy was defined as an echinocandin (micafungin [MCFG], caspofungin [CPFG], or anidulafungin), since absolute neutrophil counts were not uniformly available across sites [24]. Collected data included demographics, comorbidities (eg, diabetes, renal dysfunction with eGFR <30 mL/min/1.73 m^2^, malignancy, chemotherapy, immunosuppressive therapy), and risk factors for candidemia (eg, recent surgery, burn injuries, presence of CVCs). Microbiological data encompassed *Candida* species identification, the number of blood culture sets, the number of positive sets, and suspected sources of infection. Details regarding blood culture systems and incubation protocols in each hospital are given in Supplementary Table 1. Ophthalmologic findings were retrospectively reviewed based on the ophthalmologists’ notes. Cases were categorized as “endophthalmitis” when the diagnosis was explicitly recorded in their report.

### Outcome Measures and Statistical Analysis

The primary outcome was defined as 30-day mortality and compared between patients with high compliance (4–5 points) and those with low compliance (0–3 points). The secondary outcome was the occurrence of endophthalmitis, for which potential risk factors were subsequently analyzed.

No a priori sample size calculation was conducted, as this was a retrospective analysis including all eligible cases identified during the study period. Baseline characteristics were analyzed using the Mann–Whitney U test for continuous variables and either the chi-square test or Fisher's exact test for categorical variables, as appropriate. To reduce confounding and enhance comparability between these groups, propensity score matching was employed [25]. Age (≥75 years or less), sex, *Candida* species (*C. albicans* vs non-*albicans*), and baseline variables that showed a *P* value < .2 in univariate comparisons were included in a multivariate logistic regression model to calculate propensity scores. To minimize the risk of model overfitting, the number of covariates included in the propensity score and multivariable models was limited relative to the number of events, maintaining an events-per-variable ratio greater than 10, which is generally considered acceptable for model stability. Based on the estimated propensity scores, patients in the high compliance group were matched 1:1 with those in the low compliance group using nearest-neighbor matching without replacement. Following matching, survival outcomes were evaluated using the Kaplan–Meier analysis and compared with log-rank tests. Cox proportional hazards models were used to estimate hazard ratios (HRs) for mortality. Predictors of endophthalmitis were assessed using logistic regression. Candidate variables for secondary analysis included clearance status (persistent candidemia), central line-associated bloodstream infection (CLABSI), Candida species, and diabetes mellitus. Variable selection was guided by previous studies reporting species and CLABSI as important prognostic factors [8, 26], the clinical relevance of diabetes as a common immune-impairing condition, and the potential role of persistent candidemia in facilitating hematogenous dissemination. Statistical significance was defined as a 2-sided *P* value < .05. All analyses were performed using EZR, a graphical user interface for R (version 3.5.2).

### Ethics Approval

This study was conducted in accordance with the Declaration of Helsinki (1975, revised 2008) and approved by the Okayama University Ethics Institutional Review Board (Approval No. 2404-045). Given the retrospective design, informed consent was obtained through an opt-out process in compliance with institutional regulations.

## RESULTS

**Figure 1.**
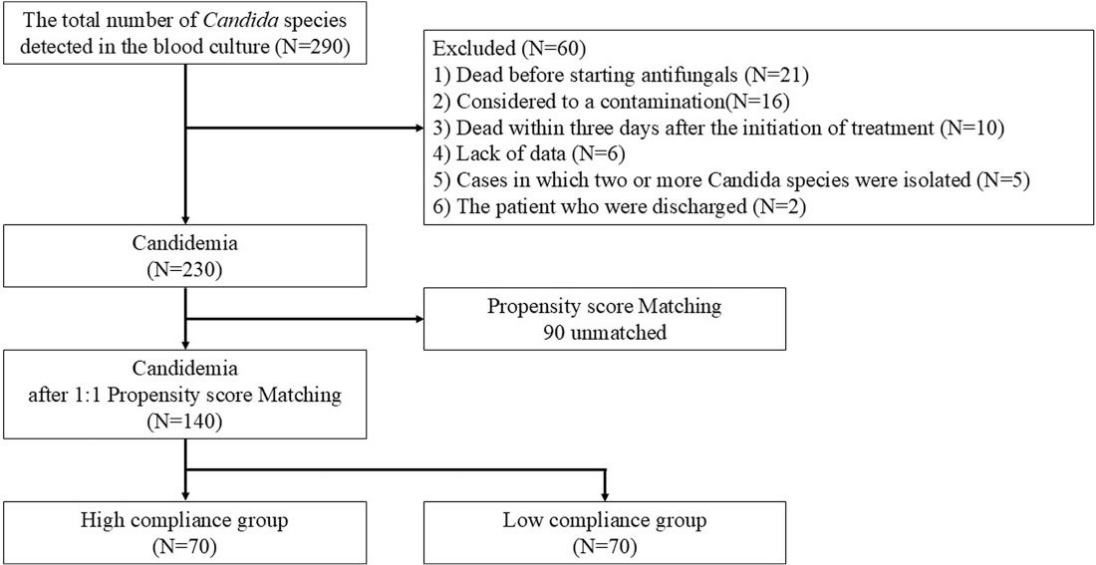
Patient enrollment flow.

During the study period, 290 patients were identified as having *Candida* species detected in blood cultures. After applying predefined exclusion criteria, 60 patients were excluded, resulting in a final analytical cohort of 230 cases. The exclusions were based on the following: death prior to the initiation of antifungal therapy (n = 21), suspected blood culture contamination (n = 16), death within 72 hours of treatment initiation (n = 10), insufficient clinical information (n = 6), and polymicrobial candidemia involving multiple *Candida* species (n = 5), and discharge prior to treatment completion (n = 2) (Figure 1).

An overview of the baseline demographic and clinical characteristics of the 230 patients is presented in Table 1. The median age was 73 years (interquartile range [IQR], 64–79), and 65.7% of the cohort were male. The majority of cases (88.3%) were classified as nosocomial candidemia. The most frequent comorbid conditions included malignancy (53.9%), diabetes mellitus (41.7%), and immunosuppressive therapy (26.1%). The central venous catheter (CVC) was the most common source of infection, identified in 66.9% of cases, followed by intra-abdominal infections (13.5%) and urinary tract infections (8.7%). Adherence to the Candida Care Bundle was generally high, with 69.5% of patients achieving a compliance score of 4 or 5 points. Regarding species distribution, *C. albicans* was the most frequently isolated organism (42.2%, 97 cases), with *C. glabrata* (23.5%, 54 cases) and *C. parapsilosis* (16.5%, 38 cases) comprising the next most common species. Initial antifungal therapy included micafungin (MCFG, 75.7%), CPFG (1.3%), amphotericin B (AMPH-B, 7.0%), fluconazole (FLCZ, 10.9%), voriconazole (VRCZ, 4.8%), and a combination of MCFG and FLCZ (0.4%). The median total duration of antifungal therapy was 20 days (IQR, 14–40). In the endophthalmitis subgroup (n = 41), the median duration was 49 days (IQR, 30–74), compared with 19 days (IQR, 15–31) in patients without endophthalmitis and 15 days (IQR, 10–26) in those without ophthalmologic evaluation. Micafungin was the most frequent initial agent (82.9%), with 38 of 41 patients (92.7%) subsequently transitioned to ocular-penetrating therapy (FLCZ, n = 33; VRCZ, n = 4; AMPH-B, n = 1).

**Table 1.** Baseline Characteristics of All Patients

	All Patients (N = 230)	High Compliance (4–5 Points) (N = 160)	Low Compliance (0–3 Points) (N = 70)	*P* Value
Age, years (Median [IQR])	73 [64–79]	73 [65–79]	72 [64–79]	.99
Sex, male, N (%)	151 (65.7%)	106 (66.3%)	45 (64.3%)	.77
In-hospital onset, N (%)	203 (88.3%)	141 (88.1%)	62 (88.6%)	1.00
Background condition, N (%)				
Malignancy	124 (53.9%)	78 (48.8%)	46 (65.7%)	.02
Diabetes mellitus	96 (41.7%)	64 (40.0%)	32 (45.7%)	.38
Immunosuppressive therapy	60 (26.1%)	38 (23.8%)	22 (31.4%)	.25
Chemotherapy	59 (25.7%)	40 (25.0%)	19 (27.1%)	.75
Recent operation	56 (24.3%)	35 (24.5%)	21 (30.0%)	.31
Chronic kidney disease	45 (19.6%)	30 (21.9)	15 (21.4)	.72
Hemodialysis	30 (13.0%)	20 (12.5%)	10 (14.3%)	.68
Burn injury	3 (1.3%)	3 (1.9%)	0 (0.0%)	.55
Origin of candidemia, N (%)				
CV catheter-related	154 (66.9%)	112 (70.0%)	42 (60.0%)	.17
Abdominal	31 (13.5%)	20 (12.5%)	11 (15.7%)	.53
Urinary tract	20 (8.7%)	13 (8.1%)	7 (10.0%)	.62
Primary	16 (7.0%)	10 (6.3%)	6 (8.8%)	.58
Other	9 (3.9%)	5 (3.1%)	4 (5.7%)	.46
*Candida* species, N (%)				
* C. albicans*	97 (42.2%)	70 (43.8%)	27 (38.6%)	.56
* C. glabrata*	54 (23.5%)	36 (22.5%)	18 (25.7%)	.62
* C. parapsilosis*	38 (16.5%)	20 (12.5%)	18 (25.7%)	.01*
* C. tropicalis*	16 (7.0%)	13 (8.1%)	3 (4.3%)	.40
* C. guilliermondii*	8 (3.5%)	6 (3.8%)	2 (2.9%)	1.00
* C. krusei*	8 (3.5%)	6 (3.8%)	2 (2.9%)	1.00
* C. dubliniensis*	6 (2.6%)	6 (3.8%)	0 (0%)	.18
Others^a^	3 (1.3%)	0 (0%)	3 (4.3%)	.03*

^a^Abbreviations: BDG, (1, 3)-β-D-glucan; CV, catheter, central venous catheter; IQR, interquartile range. Others include 2 cases of *C. famata* and 1 case of *C. lusitaniae*.

^*^Significant differences are observed in *C. parapsilosis* and others.

Patients were categorized into either the high compliance group (n = 160) or the low compliance group (n = 70). There were no significant differences between the 2 groups in terms of age, sex, or the proportion of nosocomial onset. Among underlying conditions, only malignancy was significantly more prevalent in the low compliance group compared with the high compliance group (65.7% vs 48.8%, *P* = .02). Central venous catheter was the most common source of candidemia in both groups, and *C. albicans* was the most frequently isolated species.

**Figure 2.**
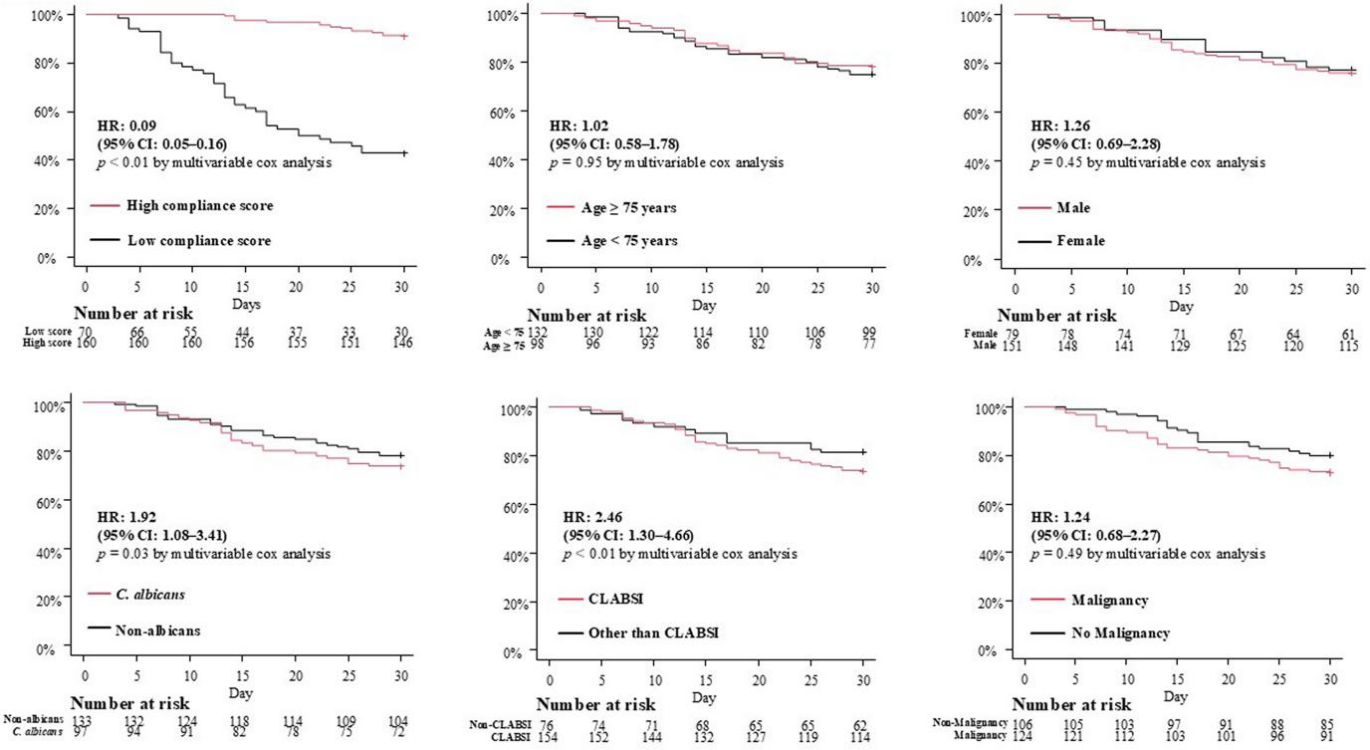
Kaplan–Meier survival curves comparing prognosis of candidemia patients stratified by potential covariates before propensity score matching. Kaplan–Meier curves showing 30-d survival stratified by the Candida Care Bundle score, age, sex, *Candida* species, presence of central venous catheter-associated bloodstream infection (CLABSI), and malignancy. Patients in the high compliance group demonstrated significantly lower 30-d mortality compared with those in the low compliance group (8.8% vs 57.1%; *P* < .01 by log-rank test).

The overall mortality rate was 23.5% (54/230 cases). Figure 2 presents Kaplan–Meier curves comparing 30-day survival stratified by Candida Care Bundle score (high compliance vs low compliance), age (≥75 years vs <75 years), sex (male vs female), *Candida* species (*C. albicans* vs non-*albicans*), diagnosis of CLABSI, and malignancy, before propensity score matching. The high compliance group demonstrated a significantly lower 30-day mortality rate compared with the low compliance group (8.8% [14/160 cases] vs 57.1% [40/70 cases]; HR, 0.09; 95% CI, .05–.16; *P* < .01). Age (HR, 1.02; 95% CI, .58–1.78; *P* = .95), sex (HR, 1.26; 95% CI, .69–2.28; *P* = .45), and malignancy (HR, 1.24; 95% CI, .68–2.27; *P* = .49) were not statistically significant risk factors for mortality, whereas *C. albicans* (HR, 1.92; 95% CI, 1.08–3.41; *P* = .03) and CLABSI (HR, 2.46; 95% CI, 1.30–4.66; *P* < .01) were identified as potentially significant risk factors.

To adjust for baseline differences between each compliance group, propensity scores were calculated using a multivariate logistic regression model, with age, sex, and *Candida* species, CLABSI onset, and malignancy as covariates. Based on these scores, patients were matched in a 1:1 ratio between the high and low compliance groups (Figure 1). After matching, no significant differences in baseline characteristics remained between the groups (Supplementary Table 2).

**Figure 3.**
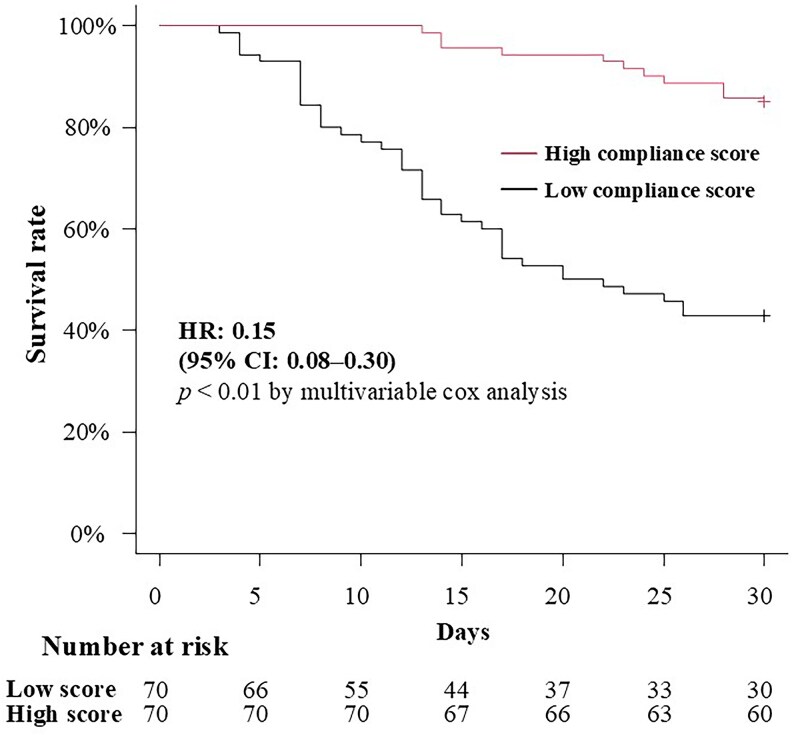
Kaplan–Meier survival curves comparing prognosis of patients with candidemia stratified by high versus low compliance with the Candida Care Bundle following propensity score matching. Kaplan–Meier survival curves comparing 30-day mortality between propensity score–matched patients with high (4–5 points) and low (0–3 points) compliance. Propensity scores were calculated using a multivariate logistic regression model including age, sex, *Candida* species, malignancy, and CLABSI. Even after matching, the high compliance group remained significantly associated with improved survival (hazard ratio: 0.15; 95% confidence interval: 0.08–0.30; *P* < .01, log-rank test).

Figure 3 illustrates the Kaplan–Meier survival curves comparing 30-day mortality between the matched groups. Even after propensity score matching, the high compliance group remained significantly associated with better 30-day mortality rate (14.3% [10/70 cases] vs 57.1% [40/70 cases]; HR: 0.15; 95% CI: 0.08–0.30). Infection with *C. albicans* (HR, 1.95; 95% CI, 1.01–3.52; *P* = .03) and the involvement of CLABSI (HR, 2.63; 95% CI, 1.35–5.12; *P* < .01) were significantly associated with increased mortality risk (Table 2).

**Table 2.** Multivariable Cox Analysis of 30-Day Mortality After Propensity Score Matching

	Hazard Ratio	95% CI	*P* Value
Age	0.90	.50 to 1.62	.71
Sex	1.27	.69 to 2.35	.44
High compliance	0.15	.08 to .30	<.01
CLABSI	2.63	1.35 to 5.12	<.01
Malignancy	1.01	.53 to 1.92	.97
*C. albicans*	1.95	1.01 to 3.52	.03

Regarding the secondary outcome, ophthalmology consultation was performed in 75.7% (174/230 cases), with the incidence rate of candida endophthalmitis at 23.6% (41/174 cases) (Supplementary Table 3). When comparing *C. albicans* with non-*albicans* species, the risk of developing endophthalmitis was significantly higher in patients with *C. albicans* infection (42.1% vs 9.2%, *P* < .01). Logistic regression analysis was performed using potential variables, such as clearance (persistent candidemia), CLABSI, *Candida* species (*C. albicans*), and diabetes mellitus. As a result, CLABSI (OR: 2.69; 95% CI: 1.08–6.70; *P* = .03) and *C. albicans* (odds ratio [OR]: 8.18; 95% CI: 4.46–19.30; *P* < .01) were found to be associated with the development of endophthalmitis (Table 3).

**Table 3.** Risk Factors of Endophthalmitis

	OR	95% CI	*P* Value
Confirmed negative	0.39	.12 to 1.32	.13
CLABSI	2.69	1.08 to 6.70	.03
*C. albicans*	8.18	4.46 to 19.30	<.01
Diabetes mellitus	1.28	.57 to 2.84	.55

Abbreviations: OR, odds ratio; CI, confidence interval.

## DISCUSSION

In this study, we evaluated the impact of the Candida Care Bundle compliance on the prognosis of patients with candidemia using propensity score matching. The overall mortality rate was 23.5% (54/230 cases), suggesting the poor prognosis of candidemia cases as has been reported elsewhere. Before matching, 69.6% of patients were classified into the high compliance group, among whom the 30-day mortality rate was significantly lower than that of the low compliance group (8.8% vs 57.1%). In the post-matching analysis as well, high compliance to the Candida Care Bundle remained significantly associated with better prognosis.

The mortality rate of candidemia remains reportedly as high as 40% [10, 27], requiring timely and appropriate management strategies for successful clinical outcomes. These include prompt source control, confirmation of blood culture clearance, appropriate antifungal selection, and administration of adequate treatment duration [20]. A set of 9 evidence-based practices, collectively referred to as the Candida Care Bundle, has been associated with improved patient outcomes when properly implemented [19, 20, 22, 28]. However, recent reports suggest that the prognostic benefit may be more pronounced when excluding the practice of oral antifungal step-down therapy [22]. Of these, we focused on 5 key components of the bundle in the present study. Patients were stratified into 2 groups according to bundle compliance, and the low-compliance group (≤3 items) exhibited a significantly higher mortality rate of 57.1% (40/70), whereas the high-compliance group (≥4 items) had a markedly lower mortality rate of 8.8% (14/160). Previous studies have suggested that factors such as age, malignancy, CLABSI, and *Candida* species may influence prognosis [10, 29, 30]. Thus, we adjusted for these potential confounders using propensity score matching to isolate the effect of bundle compliance, and found that high compliance to the Candida Care Bundle remained strongly associated with favorable outcomes. These findings underscore the importance of consistent implementation of key bundle elements, regardless of underlying patient characteristics, and further support the clinical utility of the Candida Care Bundle as a practical tool in the management of candidemia.

In cases of CLABSI, timely removal of the CVCs is considered particularly important. Numerous observational studies have demonstrated favorable outcomes associated with catheter removal, and current IDSA guidelines also strongly recommend this approach [31]. However, due to the lack of randomized controlled trials, some note a caution that definitive conclusions cannot be drawn, as existing observational studies are subject to heterogeneous patient populations, inconsistent interventions, and potential confounding factors [32]. Indeed, while some reports have shown that CVC removal is the independent factor associated with improved survival or reduced mortality (OR: 0.34; 95% CI: 0.15–0.77) [29], others have reported no significant association between early CVC removal—defined as within 48 hours—and 30-day mortality [33, 34]. Nevertheless, there is currently no evidence suggesting that CVC removal has a detrimental effect on prognosis. Therefore, based on the available data, prompt catheter removal upon the diagnosis of candidemia remains a reasonable and widely accepted clinical practice. In this study, the higher mortality observed in CLABSI may reflect not only the direct impact of catheter-related infection but also the clinical context in which these infections occur. Patients requiring central venous catheterization are often critically ill, and this factor may have contributed to the higher mortality observed in the CLABSI group. Unfortunately, due to the retrospective nature of this study, standardized severity indices such as APACHE II or SOFA scores were not consistently available across institutions, precluding appropriate statistical adjustment for baseline severity. This represents a major limitation of our analysis, and therefore caution is warranted in interpreting CLABSI as an independent risk factor for mortality. Future prospective studies incorporating validated severity scoring systems will be essential to clarify the true prognostic impact of CLABSI.

Differences in mortality risk among *Candida* species have been reported in previous studies. While some have identified *C. albicans* as a significant predictor of mortality [35], others have suggested that *C. glabrata* or *C. tropicalis* may be associated with even higher risk [10, 36]. In our study, *C. albicans* was identified as an independent risk factor for mortality after adjusting for baseline characteristics. *C. albicans* exhibits remarkable morphological and metabolic plasticity, enabling it to adapt to diverse host environments, evade immune defenses, and cause both superficial and life-threatening systemic infections. Its ability to transition between yeast and hyphal forms, acquire scarce nutrients, resist stress, and modulate host immune recognition underscores its success as a versatile human pathogen [2, 37]. Beyond its intrinsic pathogenicity, the excess mortality associated with *C. albicans* candidemia may also reflect its propensity to cause disseminated complications such as endophthalmitis [8], endocarditis [38], or osteomyelitis [39]. Moreover, biofilm formation on intravascular devices represents an additional challenge, limiting antifungal penetration and delaying eradication [40–42]. These factors, combined with host-related vulnerabilities, may explain why *C. albicans* remained an independent predictor of mortality in our cohort.

Candida endophthalmitis occurs in approximately 15%–25% of patients with candidemia [7, 26, 43], and *C. albicans* is widely recognized as a strong risk factor for its development [8]. In our study, endophthalmitis was significantly more frequent in patients with *C. albicans* infection (42.1%, 32/76) compared with those with non-*albicans* species (9.2%, 9/98). Multivariate analysis confirmed *C. albicans* as a robust independent risk factor, with an odds ratio of 9.83 (95% CI: 4.00–24.20). This may be attributed to its unique virulence traits, including yeast-to-hypha transition, strong tissue invasiveness, biofilm formation, and the ability to induce robust intraocular inflammation [6, 44, 45]. These features facilitate hematogenous dissemination and tissue penetration, making *C. albicans* more likely to invade ocular structures and elicit detectable lesions. Consistent with previous reports, CLABSI was also identified as a significant risk factor for Candida endophthalmitis in our study [8, 26]. This association is biologically plausible, as catheter-related infections often lead to persistent or high-grade fungemia due to delays in source control, thereby facilitating hematogenous seeding of the eye. In addition, biofilm formation on intravascular devices may contribute to sustained fungal release into the bloodstream, further increasing the risk of metastatic complications. Patients with CLABSI are frequently critically ill and exposed to prolonged parenteral nutrition or multiple invasive procedures, which may amplify their vulnerability to ocular dissemination. Interestingly, persistent candidemia itself was not identified as a significant risk factor for endophthalmitis in our cohort, whereas CLABSI was. One possible explanation is that intravascular catheter colonization may continuously release fungal elements at levels insufficient to be consistently detected in serial blood cultures, but sufficient to allow hematogenous seeding of distant organs such as the eye. Biofilm formation on catheter surfaces may further facilitate intermittent shedding of yeasts or hyphal fragments into the circulation, leading to ocular invasion even in the absence of overtly persistent fungemia. This pathophysiological mechanism may account for the stronger association observed with CLABSI compared with persistent candidemia. These findings underscore the importance of prompt catheter removal and early ophthalmologic evaluation in candidemia patients with suspected CLABSI.

Our findings should be interpreted considering the following limitations. First, due to the inherent limitations of the retrospective study design, we were only able to evaluate 5 key components of the bundle rather than the full 9-component protocol. Second, some cases may have been misclassified as CLABSI due to the presence of unidentified primary sources of infection, potentially leading to overestimation of CLABSI-associated risk. Third, standardized severity indices were not consistently available across institutions, precluding statistical adjustment for baseline severity and limiting a thorough evaluation of the association between disease severity and clinical outcomes. To partially mitigate this limitation, patients who died within 3 days after initiation of antifungal therapy were excluded, and clinical factors associated with severity (eg, *Candida* species, CLABSI, malignancy, and age) were included in the propensity score model. Nevertheless, the absence of standardized acute-phase severity scores remains a major limitation that may have influenced the observed association between bundle compliance and patient prognosis. Fourth, because ophthalmologic findings were extracted retrospectively from medical records, detailed information such as lesion depth, vitreous involvement, or visual acuity was not consistently available. The diagnosis solely relied on ophthalmologists’ documentation, and thus, it was not possible to distinguish between chorioretinitis—an early stage of ocular involvement without visual acuity loss—and endophthalmitis, a more advanced stage characterized by impaired visual acuity. This limitation should be considered when interpreting the frequency of ocular involvement in this study. Fifth, disseminated complications other than endophthalmitis were not systematically assessed in our study, making it difficult to fully compare the clinical manifestations between *C. albicans* and non-*albicans* species. This limitation restricts our ability to elucidate why *C. albicans* emerged as an independent risk factor for mortality. Sixth, the definition of contamination in our study was based on the clinical judgment of physicians in charge, which is an unstandardized and subjective approach. Because clinical symptoms and contextual factors may not always reliably distinguish true infection from contamination, misclassification bias cannot be fully excluded. This limitation should be considered when interpreting our findings. Despite these limitations, this study highlights the importance of compliance to the Candida Care Bundle.

## CONCLUSION

In conclusion, high compliance to key components of the Candida Care Bundle was strongly associated with improved survival among patients with candidemia, regardless of underlying patient characteristics. Routine ophthalmologic examination is warranted in all candidemia cases, especially in patients infected with *C. albicans* or those with CLABSI.

## Supplementary Material

ofaf790_Supplementary_Data

## Supplementary Data

Supplementary materials are available at *Open Forum Infectious Diseases* online. Consisting of data provided by the authors to benefit the reader, the posted materials are not copyedited and are the sole responsibility of the authors, so questions or comments should be addressed to the corresponding author.
