# Supplementary material for: Impact of Candida Care Bundle Compliance on the Prognosis of Patients With Candidemia: A Multicenter Retrospective Cohort Study With Propensity Score Matching Analysis (2016–2023)
Source: Open Forum Infect Dis. 2025 Dec 23;13(1):ofaf790. doi: 10.1093/ofid/ofaf790 (PMC12771640; doi:10.1093/ofid/ofaf790)
Supplement: ofaf790_Supplementary_Data [file ofaf790_supplementary_data.docx]

**Supplementary Table 1. Blood culture system of each facility**

|  | Blood culture bottle | Blood culture device | Days of culture |
| --- | --- | --- | --- |
| ―Okayama University Hospital  ―Tsuyama Chuo Hospital  ―Okayama Medical Center  ―Okayama Rousai Hospital  ―Okayama Red Cross Hospital  ―Takahashi Central Hospital  ―Kurashiki Medical Center  ―Tottori Municipal Hospital  ―Marugame Medical Center | BD BACTEC  BACT/ALERT  BD BACTEC  BD BACTEC  BACT/ALERT  BD BACTEC  Signal Blood Culture system  BD BACTEC  BD BACTEC | BD BACTEC FX  BACT/ALERT VIRTUO  BD BACTEC FX  BD BACTEC FX  BACT/ALERT VIRTUO  BD BACTEC FX  Signal Blood Culture system  BD BACTEC FX  BD BACTEC FX | 7  5  7  5  7  7  7  7  7 |

**Supplementary Table 2. Characteristics of propensity score-matched patients stratified by compliance with the Candida Care Bundle**

|  | All patients  (N=140) | High compliance (4–5 points)  (N=70) | Low compliance (0–3 points)  (N=70) | *p* value |
| --- | --- | --- | --- | --- |
| Age, years (Median [IQR]) | 72 [65–80] | 72 [65–82] | 72 [64–79] | 0.91 |
| Sex, male, N (%) | 89 (63.6%) | 44 (62.9%) | 45 (64.3%) | 1.00 |
| *C. albicans*, N (%) | 56 (40.0%) | 30 (42.9%) | 26 (37.1%) | 0.61 |
| CLABSI, N (%) | 89 (63.6%) | 47 (67.1%) | 42 (60.0%) | 0.48 |
| Malignancy, N (%) | 97 (69.3%) | 51 (72.9%) | 46 (65.7%) | 0.46 |

＊Abbreviations: IQR, interquartile range. CLABSI, CVC line-associated blood stream infection.

**Supplementary Table 3. Ophthalmic Evaluation**

|  | All patients  (N=230) | *C. albicans*  (N=97) | Non-albicans  (N=133) | *p* value |
| --- | --- | --- | --- | --- |
| ― Ophthalmology consultation  ― Endophthalmitis* | 174 (75.7%)  41 (23.6%) | 76 (78.4%)  32 (42.1%) | 98 (73.7%)  9 (9.2%) | 0.37  <0.01 |

＊The proportion of endophthalmitis was determined among those who underwent ophthalmologic evaluation, not the entire cohort.
